# Children with Developmental Disabilities in Low- and Middle-Income Countries: More Neglected and Physically Punished

**DOI:** 10.3390/ijerph17197009

**Published:** 2020-09-25

**Authors:** Andrea Bizzego, Mengyu Lim, Greta Schiavon, Gianluca Esposito

**Affiliations:** 1 Department of Psychology and Cognitive Science, University of Trento, 38068 Rovereto, Italy; andrea.bizzego@unitn.it (A.B.); greta.schiavon@studenti.unitn.it (G.S.); 2 Psychology Program, School of Social Sciences, Nanyang Technological University, Singapore 636921, Singapore; mengyu.lim@ntu.edu.sg; 3 Lee Kong Chian School of Medicine, Nanyang Technological University, Singapore 636921, Singapore

**Keywords:** developmental disabilities, parental involvement, low middle income countries, caregiving, child discipline, child education

## Abstract

Little is known about parenting in the context of developmental disabilities in low- and middle-income countries (LMIC), penalized by both lack of data and a research bias toward western societies. In this study, we apply data mining methods on a large (N = 25,048) dataset from UNICEF to highlight patterns of association between developmental disabilities of children and parental involvement. We focus on the co-presence of multiple disabilities and the quality of childcare in three parenting domains: discipline, caregiving, and education. Our results show that, in LMIC, children with more severe developmental conditions are also more likely to receive low-quality parental care. Specific policies of parental training are needed to improve parental practices in LMIC.

## 1. Introduction

Parenting is a common human experience and an intensely demanding one. In the United States alone, more than two million individuals become parents every year [1], spending an average of 3.86 h per day parenting young children aged under six [2]. Parenting, defined broadly as the process of rearing a child from infancy to adulthood [3], is recognized by the American Psychological Association to comprise cognitions and practices and parenting that fulfill three distinct goals: ensuring the health and safety of the child, preparing the child for life and its associated challenges, and transmitting cultural values [4]. To meet these goals of parenting, the parent engages in a variety of parenting practices, defined as concrete behavioral patterns with which the parent parents the child [5]. Parenting practices that have been heavily investigated include those related to parental nurturance and discipline [6]. For example, higher quality parental caregiving practices are linked to better attachment security [7] and fewer instances of externalizing behavior [8] in young children, thereby improving the child’s emotional health and emotional regulation skills. Similarly, disciplinary practices chosen by parents also have a significant impact on child outcomes in terms of the child’s eventual moral values [9,10] as well as internalizing and externalizing behaviors [11,12,13], which have an effect on the child’s long-term socialization skills and moral conscience.

In addition to caregiving and discipline, another significant parenting practice is in the provision of educational opportunities and resources. Although often studied in isolation, parental discipline and provision of education are related to each other [14] and have an impact on children’s language comprehension [14] and therefore eventual career prospects. Parental involvement in the child’s education is associated with higher levels of language comprehension [15] and education attainment in adulthood [16]. Altschul [17] found that, instead of a parent’s presence or time spent on the child’s education, the provision of monetary and tangible resources predict greater child achievement. As a result, parenting practices in the areas of education and resource provision are qualitatively distinct, yet related areas of investigation.

The behavioral manifestation of parenting practices in terms of caregiving, discipline and educational strategies may be moderated by the presence of several factors, such as the developmental status of the child. For a minority but still significant proportion of parents, parenting has added challenges in terms of the child’s developmental disabilities (DD), defined as conditions that arise because of delays or impairments in the child’s physical, learning, language, or behavioral adjustment [18]. Such conditions affect 52.9 million children aged below five in 195 different countries around the world [19]. In these cases, parents often report elevated parenting stress [20,21] and poor psychological well-being [22] among other negative outcomes [23]. Unfortunately, increased parenting stress is often associated with lower quality parenting and poorer child outcomes, such as inferior social competence [24]. One relevant study conducted by [25] examined relations among parenting behavior, parental stress, and child DD using a family processes approach. [25] revealed that parental stress, parenting practices, and the child’s behavioral issues due to DD, in fact, interact. For example, the association between parental stress and child behavioral adjustment has been observed among parents of children diagnosed with Autism Spectrum Disorder [26]. Negative child outcomes may in turn further affect the manner with which parents parent their child with DD, resulting in a vicious cycle that is beneficial for neither party. This cycle characterizes changes to the parents’ parenting strategies and practices, as seen in [27], which found that parents of children with DD often discipline their children more harshly than parents of typically developing children. However, much is still unknown about relations between DD and other parenting practices such as caregiving, education, and resource provision. As such, it is critical to examine how parenting practices can be influenced by, and in turn influence, the child’s developmental status and trajectory to gain insight into protecting this vulnerable population [28].

Furthermore, the generalizability of the interaction model between DD and parenting practices proposed by [25] in the three domains (caregiving, discipline, and provision of education opportunities) remains to be seen in different cultures and socioeconomic contexts, especially where parental beliefs and parenting approaches differ. As is evident by the current research literature, many studies are conducted in cultural contexts where a majority of participants belong to high-income countries with relatively homogeneous social demographic profiles (i.e., Western, Educated, Industrialized, Rich, Democratic, or WEIRD societies; [29]). Participants whose profiles do not align with WEIRD demographics are often underrepresented in the research literature, and little is known about their parenting practices, even though these parents hold beliefs and attitudes regarding DD different from those in WEIRD populations [30,31,32]. Also, parents belonging to lower socioeconomic statuses often experience greater parenting stress, which leads to poor child outcomes [33]. The need for cross-cultural studies is especially pertinent as contemporary developmental scientists look to understand child development in different cultural contexts [34], and as the global agenda shifts to improve the quality of life for children around the world [35], especially those in low- and middle-income countries (LMIC), where the prevalence of DD is likely higher than in high-income countries [36]. Therefore, there is a need to investigate parenting practices across different cultures to obtain findings that are more ecologically valid and generalizable.

### Aim of This Study

Child DD is associated with various negative parenting behaviors, such as harsh discipline [27]. We hypothesized that the severity of child DD, and by extension the level of specialized knowledge, skill, and resources required to care for a child with DD [37], would influence relations between child DD and parenting strategies. Therefore, we investigated associations between the severity of developmental disabilities and three key parenting behaviors: caregiving, discipline, and opportunities for education. Specifically, the study aimed to answer three questions:

1. On the caregiving domain: Are children with more severe DD more likely to experience neglect (i.e., lower levels of caregiving) by their caregivers?
2. On the discipline domain: Are children with more severe DD more likely to suffer harsher discipline (i.e., more physical punishment)?
3. On the education domain: Are children with more severe DD more likely to be offered fewer educational resources by their caregivers?

In answering these questions, we also considered education level of the main caregiver, wealth of the household, the Human Development Index (HDI) of the country and gender of the child, to understand if any of these variables moderate relations between the child’s severity of DD and caregivers’ parenting. Due to scarcity in relevant literature on associations between child SDD and parenting behaviors in LMIC, our study is exploratory in nature. Starting from a large (N = 25,048) cross-national data set of children in LMIC and adopting a data-driven approach, we aimed to uncover patterns and provide directions for future research. We used the “Ten Questions Questionnaire” [38] to compute an index of the severity of the disability condition and categorize children into those with a Severe Developmental Disability (SDD) and those with Mild or No Disability (MND) and compare the incidence of parental practices in the two groups.

## 2. Methods

### 2.1. Dataset

Data for this study were extracted from the 2005–2007 Multiple Indicator Cluster Survey (MICS) [39]. The MICS is an international nationally representative household survey developed and supported by UNICEF. The MICS collects data on more than 200 key indicators of physical and social conditions that impact the well-being of children, women, and men. The MICS is divided into questionnaires that address macro-areas (e.g., Child development, Quality of the Housing Environment), and modules that investigate specific aspects of questionnaire macro-areas (e.g., modules on Discipline in “Household Questionnaire”, modules on Caregiving in the “Questionnaire for Children Under Five”). Each module focuses on specific members of the household within a specific age range. Subsets of MICS indicators (described below) were selected from pertinent modules to represent each parenting domain. Target households in the MICS are randomly selected following a hierarchical process, starting from census enumeration areas to segments in each area to particular households within each segment [40].

#### 2.1.1. Participants

Participants consisted of respondents to questionnaires specified in sections below. However, the target age ranges of each questionnaire differ; for example, the Child Disabilities module targets children aged 2 to 9 years, whereas indicators addressing the parenting strategies of interest (i.e., caregiving, discipline, and education) target children aged below 5 years. To facilitate analyses investigating relations between child disability and parenting, the common participant pool between different questionnaires was used (i.e., children aged between 2 and 5 years). As a household might host multiple families, we randomly sampled one child for each household and that child’s caregivers.

The final dataset is composed of 25,048 children (Males = 12,816; Age (months): Mean = 40.7, SD = 9.8) from 13 LMIC (see Table 1). Ethics approvals were handled in each site in which data were collected. The study was approved by the Internal Review Board of the Nanyang Technological University.

#### 2.1.2. Child Developmental Disabilities

**Figure 1:**
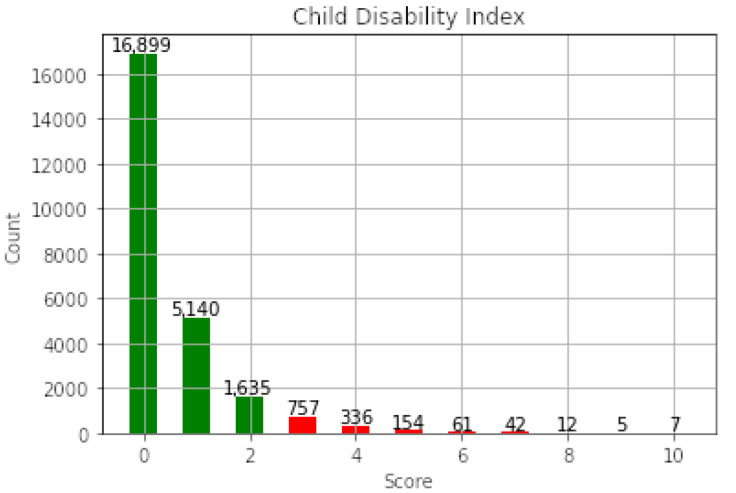
Distribution of the Child Disability Index (CDI). In red are the children that are categorized with a Severe Developmental Disability (SDD).

The Child Disabilities module of the MICS “Household Questionnaire” was used to derive a Child Disability Index (CDI) for each child. The Child Disabilities module is based on the “Ten Questions” that screen (“Yes/No”) for any cognitive, language, sensory, or motor impairment in a child [27,38,41] (see Table 2). The Child Disabilities module is answered by the mother or primary caregiver of the child. As recommended by UNICEF for inclusion into MICS to obtain an internationally comparable index of child disability [42], the “Ten Questions” report the presence of a disability in each domain, but not the severity of any disability, which is left to additional appropriate diagnostic methods [43,44]. We used the “Ten Questions” to estimate a quantitative Child Disability Index (CDI), the count of answers associated with all disabilities out of 10 (Figure 1). Thus, greater values of the CDI indicate multiple disabilities, possibly indicating more severe conditions.

### 2.2. Parenting Practice

We investigated associations between the CDI and parenting practices focusing on three domains included in the MICS: caregiving, discipline, and education. We aimed to assess whether the presence of multiple disabilities, and, thus, a more severe overall disability condition, is associated with different probabilities for children receiving good quality parental care. In the following subsections we describe, for each domain, the indexes we derived to quantify quality of the parental care.

The set of modules available in the MICS differs for each country: the modules used to compute the parenting categories were not available for all countries considered. In addition, for some households there were missing or incomplete data. Consequently, the actual sample sizes differ for each parenting practice.

#### 2.2.1. Caregiving

We investigated whether the presence of multiple disabilities is associated with a higher probability that caregivers avoid caregiving. The Caregiving module from the “Questionnaire for Children Under Five” questionnaire of the MICS investigates who in the household administers six main caregiving activities with the child. Three activities are related to the Cognitive development: “Read Books”, “Tell Stories”, “Spend Time/Name, Count, Draw”, and three refer to the socio-emotional development: “Sing Songs”, “Take Outside”, “Play”. For each activity, three questions ask whether the mother, the father, and other caregivers engage in the activity. We created three caregiving categories: (a) Cognitive, (b) Socioemotional, and (c) all activities. To quantify negative parental practices for each child caregiving category, we identified children who are neglected (no activity performed by any caregiver). A similar categorization was done for each caregiver type, identifying children who are neglected by the mother, father, and other caregivers. The Caregiving module has been used previously to examine caregiving behavior in parents [45].

#### 2.2.2. Discipline

We investigated whether multiple disabilities are associated with a higher probability that caregivers resort to severe discipline strategies. The Discipline module of the “Household Questionnaire” investigates which types of discipline strategies are used with a selected child in the household. Discipline questions are derived from the Parent-Child Conflict Tactics Scale [46] and based on the WorldSAFE survey questionnaire [47]. We used the four discipline categories [48,49] to assess the adoption of discipline practices: (a) Non-Violent, (b) Psychological Aggression, (c) Physical Violence, and (d) Severe Physical Violence. The Parent-Child Conflict Tactics scale from which the Discipline model has discriminant and construct validity [46]; the WorldSAFE questionnaire is based on ecological and social frameworks [50,51,52].

#### 2.2.3. Education

We investigated whether multiple disabilities are associated with a lower probability that caregivers offer educational opportunities to the child. Two questions from the “Child under 5” questionnaire were used: how many books are available for the child and whether or not the child attends an early education program. Two separate categories to quantify the parental education practices were created: (a) children whose caregivers provide no books and (b) children who attend no early education program. These items are relevant to the domain of provision of educational resources as part of in-home stimulation [53,54]. The questions have been used previously to examine child education [55].

#### 2.2.4. Moderators

In investigating associations between developmental disabilities and parenting, we took into account the possible influence of four moderators: education of the main caregiver, wealth of the household, socioeconomic level of the country, and gender of the child. The education of the main caregiver (No education, Primary, Secondary or Higher) was derived from two MICS indicators: “Ever attended school” and “Highest level of education attended” (Household Questionnaire). An additional education level is reported in the MICS (Preschool) but, due to the small number of caregivers in this category (n = 27), this category was discarded. The wealth of the household (Poorest, Poor, Middle, Rich, Richest) was derived from the “Wealth Index Quintile” indicator (Household Questionnaire). The socioeconomic level of the country was quantified by the 2006 Human Development Index [56]. The gender of the child was derived from the “Gender of child” indicator (Questionnaire for Children Under Five).

### 2.3. Data Analysis

A multivariate logistic regression was conducted on each parenting practice to evaluate the contribution of the CDI (score from 0 to 10) and moderators to predict the outcome of the parenting practice. To better investigate the association between CDI and the parental practices that resulted significantly associated, we dichotomized the CDI to assess whether parenting practices differ for children with multiple disabilities and children with few or no disabilities. Children with CDI≥3 (at least 3 disabilities observed) were categorized as children with Severe Developmental Disabilities (SDD, N=1,374), and children with CDI<3 were categorized as children with Mild or No Disability (MND, N=23,674). This threshold was empirically set as a trade-off between two requirements: (a) a lower threshold value to have a statistically relevant number of children in the SDD category and (b) a higher threshold value to avoid including in the SDD category children with mild conditions. We note, however, that the number of children in the MND and SDD categories is still highly imbalanced: to obtain more conservative results, we adopted the bootstrap procedure, described below.

For each parental practice that emerged associated with the CDI in the logistic regression analyses, we then conducted Pearson’s $\chi^{2}$ tests of association between the SDD and MND categories and the outcome in terms of the parenting practice. To quantify the effect of the disabilities on each parenting variable, we report the Odds Ratio of being exposed to a negative parental practice for SDD children with respect to MND children ($OR_{SDD/MND}$): values greater that 1 indicate that SDD children are more likely than MND children to be exposed to the negative parental practice. In doing so, we also investigated the influence of each moderator: the Pearson’s $\chi^{2}$ test and computation of the $OR_{SDD/MND}$ was conducted on each category of the moderator to allow the observing of differences between the categories. From the HDI (which is a continuous index varying from 0 to 1) we obtained two categories of countries: Low HDI (HDI<0.5) or High HDI (HDI≥0.5). For each moderator, we apply the Benjamini–Hochberg correction for False Discovery Rates to account for multiple comparisons.

#### Bootstrap $\chi^{2}$ Test

Due to the imbalance in the number of children in the MND and SDD groups, we adopted a bootstrapped version of the $\chi^{2}$ test. Given NSDD as the number of SDD children in the dataset for a given parenting domain, we randomly selected the same number of MND children as there were SDD children (NSDD ) and computed a $\chi^{2}$ test on the dataset composed of all SDD children and selected MND children, obtaining $\chi^{2}$, p, and a Cramer’s V estimate. This procedure was repeated 1000 times, each time selecting a random subset of MND children. After the 1000 iterations, we obtained bootstrapped $\chi^{2}$ test results by computing the median values of the computed $\chi^{2}$s, ps, and a Cramer’s V estimates. Statistical significance was evaluated on the median $\chi^{2}$ (α=0.05). The *p*-values computed by the bootstrap procedure are higher than standard Pearson $\chi^{2}$ test and, as a consequence, ensure more conservative results.

## 3. Results

The results from the multivariate analyses (Table 3) indicate that the CDI significantly predicts the outcome of being neglected in terms of cognitive caregiving activities and the exposition to severe physical punishment.

Regarding being neglected in terms of cognitive caregiving activities, all moderators except the gender of the child are also significantly associated. Regarding the exposition to severe physical punishments, only the Wealth of the Household and the HDI of the country are significantly associated.

We then categorized children into the MND and SDD categories. All moderators, except the gender of child, are associated with the incidence of SDD (Table 4).

**Figure 2:**
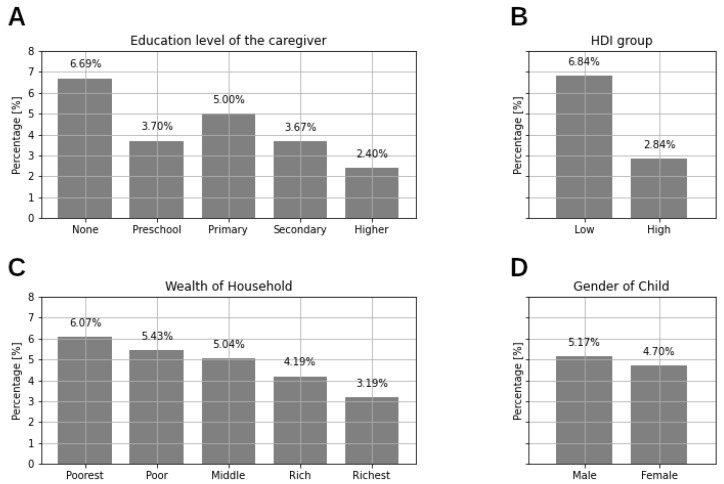
Percentages of SDD children for each category of the moderators: (**A**): Education level of the caregiver; (**B**): HDI group; (**C**): Wealth of the household and (**D**): Gender of the child.

Education of the child’s main caregiver is significantly associated with the incidence of SDD: the percentage of children with SDD decreases with increasing education level of the child’s caregiver (from 6.69% for no education to 2.40% for higher education, see Figure 2). Wealth of the household is also significantly associated with the incidence of SDD: the percentage of children with SDD decreases with increasing wealth of household (from 6.07% for the poorest households to 3.19% for the richest, see Figure 2).

Country HDI is also significantly associated with the incidence of SDD; the incidence of SDD in Low HDI countries (6.84%) is higher than the incidence of SDD in High HDI countries (2.84%).

### 3.1. Caregiving and Developmental Disabilities

The $OR_{SDD/MND}$ of being neglected in terms of cognitive caregiving activities is 1.515 for caregiving and socioemotional activities and 1.533 for all activities (first row of Table 5). We also considered how the type of caregiver moderates this association: the difference between SDD and MND remains significant only when we consider mothers, but not fathers or other caregivers. The percentages of children neglected by fathers are the highest for both SDD and MND children. When considering education level of the caregiver a significant association is found for Primary education level ($OR_{SDD/MND}$ = 1.569). Overall, we observe that the percentages of neglected children for both SDD and MND categories decrease from No education (${\%}_{MND}$ = 34.6; ${\%}_{SDD}$ = 36.3) to Higher (${\%}_{MND}$ = 4.3; ${\%}_{MND}$ = 12.5) education levels.

When considering the wealth of the household, a significant association is found for the Poorest households ($OR_{SDD/MND}$ = 1.569). Again, the percentages of neglected children for SDD and MND categories decrease from Poorest to Richest.

High HDI countries show higher $OR_{SDD/MND}$ than low HDI countries (High HDI: $OR_{SDD/MND}$ = 1.741; Low HDI: $OR_{SDD/MND}$ =1.222), but also lower percentage of neglected children. The association between the disability category and being neglected in terms of cognitive caregiving activities is significant only for High HDI countries.

### 3.2. Discipline and Developmental Disabilities

Among children who receive physical punishment, SDD children are more likely to receive severe physical punishment than MND children ($OR_{SDD/MND}$ = 2.074, Table 6). The wealth of the household appears to have a strong effect in moderating the association between SDD and exposure to severe physical punishment: no association results remained significant (after false discovery rate correction) when partitioning the dataset according to the wealth score of the households. The association is significant for Low HDI countries (OR = 1.626), but not for High HDI countries.

## 4. Discussion

The preliminary analysis of population characteristics and the incidence of SDD has shown that SDD is generally significantly more prevalent among children (1) of caregivers with lower education levels, (2) living in poorest households and (3) residing in countries with lower HDI; the latter two conditions are reflective of generally lower socio-economic statuses (SES) [56,57].

This is congruent with findings from other studies across the world that have found increased incidence of SDD in households reporting lower income [58,59,60,61]. In addition, it is also commonly observed that individuals from an economically poorer society face greater barriers to accessing education [62,63], and may explain the correlations between caregiver education, wealth, HDI, and child SDD. It is theorized that higher child SDD incidence may be reported among these populations due to two reasons: firstly, that prenatal and postpartum exposure to adversity in terms of resource scarcity and other psychosocial hazards may impair typical child development [64]; secondly, that uneducated caregivers may not be sufficiently knowledgeable to model adaptive behaviors for optimal child development [65].

### 4.1. Caregiving and Developmental Disabilities

Multivariate and odds ratio analyses have shown that children with SDD are more likely to be neglected by their (maternal) caregivers, while paternal and other caregivers tended to show greater negligence for all children regardless of developmental status. The difference in caregiving and neglect between parents can be explained by traditional gender roles in parenting a child, where maternal figures are more often associated with direct parenting while paternal figures are associated with providing material and financial support to the family unit [66]. The specialization of parenting roles by gender indicates that fathers are less likely to be able to actively parent their child and therefore reflect greater negligence. As for the difference in caregiving provided by mothers for children with SDD and MND, multiple studies making use of a variety of methodologies consistently found that child disability was a risk factor for caregiver neglect [67,68,69,70], up to three times that of a typically developing child [71]. A potential reason for this discrepancy may lie in the difficulty in sustaining a warm responsive parenting style towards children with SDD [72], as developmental disabilities often present with signs that discourage active caregiver-child interaction such as low rates of social initiation and avoidant gaze among other behavioral characteristics [73]. Another related study conducted by [74] has confirmed that mothers’ maintenance of scaffolding and maternal behavior was directly related to the child’s receptiveness.

In addition to developmental status, other factors such as the education of the caregiver and SES contributed significantly to the difference in the level of neglect. Specifically, in terms of caregiver education, the difference in level of neglect arose among caregivers who had an intermediate level of education, where SDD children of these caregivers tended to be more neglected with respect to cognitive activities. This finding is contrary to what was reported by [75,76], which, on a tangential level, found no significant effect of education level on maternal perception of the parent–child relationship. We posit that this U-shaped relationship between education level of caregivers and level of caregiving may be due to the fact that caregivers with an intermediate level of education are able to appreciate the importance of child caregiving, but are inadequately equipped to care for a child with SDD, therefore contributing to the significant difference in caregiving. Of course, further studies would need to be conducted to confirm the nature of such a relationship in the context of child disability.

Interestingly, when examining the effect of SES on caregiving practices, caregivers from poorer households or from the higher HDI countries were more likely to show differences in cognitive caregiving based on child DD, showing seemingly contrasting directions of influence on caregiving. In terms of household wealth, this difference may be due to the struggle between spending time to maintain financial income and direct caregiving. Caregivers of poorer households may hold low-skilled jobs that have irregular working schedules or long working hours [77]. These caregivers may therefore be less able to be involved in caregiving [78,79], especially for those with low incomes [80]. On the other hand, in terms of national HDI, this difference may be due to burdens of caregiving that are unrelated to financial ability. Green [75] proposed two potential sources of burden on parenting a child with SDD, which may have implications on the eventual level of caregiving, namely objective burden (in terms of socio-structural constraints) and subjective burden (in terms of emotional distress). Following this model of parenting burden, higher national HDI should be correlated with higher levels of caregiving, as greater per capita income, education and lifespan implies greater financial, educational and healthcare resources, thereby reducing the amount of objective burden experienced by these caregivers. However, it must also be noted that one of the most significant sources of caregiving burden also come from social withdrawal and the presence of social stigma [81], which contribute to both objective and subjective burden respectively. From the perspective of caregivers of higher wealth, we posit that these caregivers in High HDI countries may in fact face greater social stigma and have to withdraw from a greater range of social activities, due to greater social capital granted those who are more financially wealthy and educated [82], resulting in a greater net increase in burden and therefore show more neglect of their child with DD. When viewed together, there may be an interaction effect between personal household wealth and national HDI on caregiving practices that is yet to be uncovered. Further studies that investigate both these factors in conjunction with child disabilities will be warranted to confirm these findings.

### 4.2. Discipline and Developmental Disabilities

Multivariate analysis revealed that children with SDD are generally more harshly punished physically compared to children with MND. The main finding is corroborated by reviews from [83,84], where it was found that children with SDD tended to be punished more harshly by their parents than children with MND. Deater-Deckard [85] posited that this trend may be due to the heightened challenges of parenting a child with DD, leading to greater levels of parenting stress [20,21,86] and thereby increasing the likelihood of reactive or angry parenting strategies.

However, these differences in discipline strategies are significantly contributed to by the household wealth and low national HDI. After grouping by household wealth and statistical correction, the differences in child DD on discipline strategies ceased to remain. Our findings support that of [87], where it was found that parents who were economically poorer tended to use more physical punishment, but were in disagreement with [88], which found less educated individuals to be more accepting of physical punishment of children. In our case, educational level did not have a significant effect on the discipline strategy chosen.

### 4.3. Education and Developmental Disabilities

It was revealed from the initial multivariate analysis that there are no significant differences between children with SDD and MND and their opportunities to access education, in terms of both possession of books and opportunities for early education. As previously mentioned in the literature review, provision of educational resources is a qualitatively different domain of parenting strategy as compared to caregiving and discipline, and may partially explain the lack of significant findings between child DD, other caregiver and child characteristics, and the access to education. In fact, according to a model of parental decision making in the context of education proposed by [89], it was the parental perception of teacher invitations for at-home educational resources that had the largest impact on the level of eventual involvement and provision of educational resources, rather than the parents’ view of their efficacy or currently available resources. This may be reflected in our study as a lack of significant relationship between child DD and caregiver educational strategies, as parenting practices in this domain may have been informed by factors that fall outside of the consideration of this study.

### 4.4. Limitations

Our study contains several limitations.

Firstly, our methodology did not begin with a formal assessment of developmental disability as defined by either the DSM-5 or the ICD-10. While this approach would be more demanding in terms of manpower and logistics, formal assessment and diagnosis of disabilities would allow for an internationally-recognized definition of disability. This would be critical as a basis for categorizing the participants, and finer differentiation between different types of developmental disabilities and severity of disability, especially as parenting strategies may differ based on whether the child has a mild or severe form of developmental disability [90]. While not a substitute for a formal clinical examination, the Ten Questions screen is sensitive in detecting severe disabilities among children aged two to nine, and shows no bias in detecting disability between girls and boys [38]. However, the Ten Questions tend to produce some false positives, where positive cases did not eventually qualify for a formal diagnosis (78%); only 70% of these children had mild disability or other health conditions, whereas 30% had no disability [38]. The implication of these false positives may in fact strengthen the reported relations between child SDD and parenting strategies in the results, as it is more likely that significant differences were contributed by a smaller sample than is calculated.

Secondly, as the modality of data acquisition is based on survey questionnaires, data related to sensitive topics such as instances of severe physical violence or even indicators of developmental delays may be subjected to issues related to social desirability [91] due to the stigma households may face in bringing up a child with a disability [92]. The nature of the MICS and data obtained had also limited the range of statistical analyses that could be performed. For example, MICS did not disambiguate between biological or adoptive parents, nor ascertain the presence of both mother and father in the household, giving rise to the possibility of single-parent households being compared with two-parent households. While “Mother” and “Father” therefore can be interpreted by the respondent as the people who serve in the social role of parents, regardless of their biological relation to the child [93], factors such as household wealth may be significantly affected by the size of the household and the presence or absence of mother or father. The difference between a single- and two-parent household may be significant as the number of possible income earners and active caregivers are different. Additionally, precise data measuring socioeconomic statuses of each household is also not available in MICS. Instead, household wealth was used, which is a valid measure of household SES in LMICs [94]. Nonetheless, with urban-rural disparities and different standards of health and educational outcomes in LMICs compared to higher-income countries, more comprehensive economic and development factors may need to be implemented in future studies in order to allow for global comparisons.

Thirdly, as the MICS is a cross-sectional study with no longitudinal follow-up, causality cannot be ascertained between the significant factors discussed above, or if another external factor not examined in the MICS (such as caregiver disability; [67]) was responsible for the relations between child disability and parenting approaches.

Lastly, although the focus of the present paper examined the relationship between the severity of child disability and parenting practices, it would be worthwhile to also examine the relationship between different types of disability and parenting strategies. As child disability consists of a broad range of conditions and presenting symptoms from physical or intellectual disabilities to sensory impairment, the nature of the disability and the needs of the child would undoubtedly differ. While this investigation is beyond the scope of this paper, a separate analysis differentiating between physical and intellectual child disabilities is ongoing [95].

Future studies expanding upon this work may wish to consider implementing formal assessment processes and other modes of data acquisition (such as observation and behavioral coding over a longer time period) to address these limitations.

### 4.5. Implications

Despite the limitations of this research discussed above, the findings were able to shed first light on the parenting strategies most associated with severe child DD in LMICs. While caregivers in LMICs generally tended to show the same patterns of parenting behavior as caregivers in higher income countries, it is noted that socioeconomic factors such as education attainment and HDI also play a significant role in mediating the known relationships between severe child DD and chosen parenting strategies. Therefore, clinicians and healthcare professionals in LMICs may need to consider interventions that lie beyond direct clinical intervention, and make use of a mix of psychoeducation and medical social work in order to decrease the stigma, as well as social and financial burden faced by parents in LMICs. 

## Figures and Tables

**Table 1:** Partitioning of the sample of the study into the 13 Low and Middle Income Countries, with the Human Development Index (HDI) of each country.

Country	N	$N_{\mathrm{MND}}$	$N_{\mathrm{SDD}}$	HDI
Albania	668	649	19	0.709
Belize	394	357	37	0.676
Cameroon	2818	2676	142	0.47
Central African Republic	4098	3694	404	0.328
Djibouti	1054	860	194	0.414
Georgia	1093	1048	45	0.712
Ghana	1725	1662	63	0.518
Lao PDR	2086	2045	41	0.511
Mauritania	3946	3741	205	0.479
Mongolia	1883	1832	51	0.66
Suriname	1176	1120	56	0.682
Uzbekistan	2457	2443	14	0.635
Yemen	1650	1547	103	0.478

**Table 2:** “Ten Questions” that screen for child disabilities. The Answer Indicating Disability column indicates the answer associated with a potential disability.

	Question	Answer Indicating Disability
Q1	Any serious delay sitting, standing or walking?	Yes
Q2	Does she/he have difficulty seeing in daytime or nighttime?	Yes
Q3	Does she/he appear to have difficulty hearing?	Yes
Q4	When you ask her/him to do something, does she/he seem to understand what you say?	No
Q5	Does she/he have difficulty walking or moving?	Yes
Q6	Does she/he have fits, become rigid or lose consciousness	Yes
Q7	Does she/he learn to do things like other?	No
Q8	Can she/he says recognizable words?	No
Q9A	Can she/he name at least one object? (For 2 years old children)	No
Q9B	Is her/his speech in any way different from normal? (For children older than 2 years)	Yes
Q10	Compared to other children does she/he appear mentally backward, dull or slow?	Yes

**Table 3:** Coefficient of the CDI and moderators of the logistic regressions with *p*-values.

Parenting Category	Parenting Outcome	CDI	Education Level	Wealth	HDI of Country	Gender of Child
Caregiving	No Cognitive Activities	0.053 (*p* = 0.00047)	−0.354 (*p* < 0.00001)	−0.253 (*p* < 0.00001)	−0.167 (*p* < 0.00001)	−0.025 (*p* = 0.10754)
	No Socioemotional Activities	0.009 (*p* = 0.68639)	−0.253 (*p* < 0.00001)	−0.212 (*p* < 0.00001)	−0.471 (p < 0.00001)	−0.014 (*p* = 0.57013
	No activities	0.019 (*p* = 0.45525)	−0.257 (*p* < 0.00001)	−0.261 (*p* < 0.00001)	−0.469 (*p* < 0.00001)	0.009 (*p* = 0.74107)
Discipline	Only non violent strategies	−0.050 (*p* = 0.2364)	0.138 (*p* = 0.00642)	0.084 (*p* = 0.04306)	0.189 (*p* = 0.00004)	0.024 (*p* = 0.52637)
	Psychological Aggression	−0.029 (*p* = 0.25746)	−0.173 (*p* < 0.00001)	0.014 (*p* = 0.61869)	−0.356 (*p* < 0.00001)	0.005 (*p* = 0.83823)
	Physical Punishment	−0.038 (*p* = 0.12295)	0.083 (*p* = 0.007)	−0.048 (*p* = 0.06983)	−0.767 (*p* < 0.00001)	−0.033 (*p* = 0.16303)
	Severe physical punishment	0.161 (*p* < 0.00001)	−0.027 (*p* = 0.50456)	−0.147 (*p* = 0.00006)	−0.337 (*p* < 0.00001)	−0.054 (*p* = 0.09938)
Education	Never attended early education programme	−0.005 (*p* = 0.82825)	−0.212 (*p* < 0.00001)	−0.730 (*p* < 0.00001)	−0.604 (*p* < 0.00001)	−0.032 (*p* = 0.15417)
	No books	0.016 (*p* = 0.43611)	−0.626 (*p* < 0.00001)	−0.572 (*p* < 0.00001)	−0.961 (*p* < 0.00001)	0.036 (*p* = 0.04909)

**Table 4:** Results of the bootstrap Pearson $\chi^{2}$ test of association between incidence of SDD and the moderators groups (N=23,696, $N_{SDD}=1170$).

	$\chi^{2}$	*p*	Cramer’s V
Education level of the caregiver	50.017	<0.00001	0.146
Wealth of Household	28.764	<0.00001	0.111
HDI	228.837	<0.00001	0.313
Gender of child	1.343	0.24652	0.024

**Table 5:** Percentages of MND and SDD children neglected in terms of cognitive caregiving activities with $OR_{SDD/MND}$ and statistics of the $\chi^{2}$ tests for different types of moderators considered. *N* and $N_{SDD}$ in the second and third columns indicate the total number of children and number of children in the SDD category in the dataset used to compute the bootstrap $\chi^{2}$ tests.

	*N*	$N_{\mathrm{SDD}}$	${\%}_{\mathrm{MND}}$	${\%}_{\mathrm{SDD}}$	${\mathrm{OR}}_{\mathrm{SDD}/\mathrm{MND}}$	$\chi^{2}$	Cramer’s V	*p*-Value	Corrected *p*-Value
All Caregivers	24,300	1226	22.7	30.8	1.515	20.44	0.091	0.000006	0.000006
Mothers	24,300	1226	44.8	53.5	1.416	17.99	0.086	0.000022	0.000066
Fathers	24,300	1226	73.9	74.6	1.036	0.26	0.010	0.611906	0.611906
Others	24,300	1226	57.5	60.4	1.126	2.06	0.029	0.150852	0.226278
No education	7149	490	34.6	36.3	1.081	0.29	0.017	0.593090	0.593090
Primary	7980	409	23.9	33.0	1.569	7.78	0.098	0.005290	0.021160
Secondary	7575	285	14.0	20.7	1.597	3.96	0.083	0.046576	0.093152
Higher	1262	32	4.3	12.5	3.173	0.87	0.116	0.351566	0.468755
Poorest	5911	359	31.6	41.2	1.519	6.96	0.098	0.008341	0.041705
Poor	5236	283	25.6	29.3	1.205	0.89	0.040	0.345892	0.345892
Middle	4795	239	21.9	30.5	1.564	3.89	0.090	0.048556	0.080927
Rich	4265	179	17.7	22.3	1.339	1.12	0.056	0.288972	0.345892
Richest	3780	119	12.8	24.4	2.198	4.71	0.141	0.029952	0.074880
Low HDI	12,822	900	28.3	32.6	1.222	3.59	0.045	0.058071	0.058071
High HDI	11,478	326	16.6	25.8	1.741	7.73	0.109	0.005430	0.010860

**Table 6:** Percentages of MND and SDD children exposed to severe physical punishment, with $OR_{SDD/MND}$ and statistics of the $\chi^{2}$ tests. *N* and $N_{SDD}$ in the second and third columns indicate the total number of children and number of children in the SDD category in the dataset used to compute the bootstrap $\chi^{2}$ tests.

	*N*	$N_{\mathrm{SDD}}$	${\%}_{\mathrm{MND}}$	${\%}_{\mathrm{SDD}}$	${\mathrm{OR}}_{\mathrm{SDD}/\mathrm{MND}}$	$\chi^{2}$	Cramer’s V	*p*-Value	Corrected *p*-Value
All Caregivers	5148	310	27.6	44.2	2.074	17.51	0.168	0.000029	0.000029
Poorest	1168	82	32.2	41.5	1.490	1.29	0.089	0.256450	0.256450
Poor	1101	65	27.8	46.2	2.226	4.00	0.175	0.045596	0.113990
Middle	1011	47	27.4	53.2	3.013	5.34	0.238	0.020783	0.103915
Rich	915	44	25.8	40.9	1.988	1.85	0.145	0.173603	0.217004
Richest	694	25	20.6	44.0	3.023	2.30	0.214	0.129558	0.215930
Low HDI	3038	254	36.2	48.0	1.626	6.79	0.116	0.009165	0.018330
High HDI	2110	56	16.0	26.8	1.925	1.33	0.109	0.249561	0.249561
